# The Association of Southern Medical Colleges

**Published:** 1892-12

**Authors:** 


### THE ASSOCIATION OF SOUTHERN MEDICAL COLLEGES.

In pursuance of a call issued to all the medical schools in the South, there was established an organization with the above name at Louisville, Kentucky, on November 16, of the current year. The organizing convention was announced to take place, for the sake of convenience, in the place and at the time of the annual meeting of the Association of Southern Surgeons and Gynecologists, and during this first meeting it was decided to continue to assemble in connection with the latter Society, for the same reason. As convened, the Association was composed of delegates from the following institutions (save in several instances where representation was by letter): Medical Department of the University of Nashville and Vanderbilt University, University of Louisville, Medical Department of the University of Tennessee, Southern Medical College of Atlanta, Chattanooga

Medical College, Memphis Medical College, Charleston Medical College, Medical Department of Tulane University, Kentucky School of Medicine, Medical Department of the University of Georgia, Mobile Medical College, Medical Department of the University of Texas, Knoxville Medical College, Little Rock Medical College, Atlanta Medical College, Louisville Medical College, and Marion Sims Medical College. As stated in the regulations for the maintenance of the Association, the objects of the organization are "to cultivate closer and more intimate relations between medical colleges and to elevate the standard of medical education by requiring a more thorough preliminary training and an incaeased length of medical study."

While the delegates present attended the convention with power to act for their respective schools in general matters connected with the elevation of medical education, it was deemed best to refer the actions of the Association to the different schools for ratification, this of course being necessary for continuation of membership in the Association. It is to be understood, further, that the ratification of these measures does not prohibit more stringent requirements on the part of such institutions as may desire them. Aside from the organization of the Association, the most important matter was the adoption of the following requirements for matriculation and graduation in the schools composing its membership. [Rules and Regulations published elsewhere in this issue.—D.]

The Medical Department of the University of Texas was represented by Prof. J. E. Thompson, in place of Prof. Allen J. Smith, who was prevented from attending because of the death of a relative. Prof. Thompson advocated the above measures, with the exception of the clause permitting the continuation of an unqualified student to the end of the first course of lectures before requiring that his literary qualifications be established. He states that the liveliest interest was manifiested in the convention as to the possibility of a reduction of the fees in the Medical Department of the University of Texas [as advocated by this journal in November issue.—D.], particularly in view of the fact that the action of the convention will probably lower decidedly the number of Texas students who annually leave the State for medical education, and turn them toward the doors of their own State school. A. J. S.

